# Health and Lifestyles Factors Associated With Osteoarthritis among Older Adults in Portugal

**DOI:** 10.3389/fmed.2017.00192

**Published:** 2017-11-08

**Authors:** Natália Duarte, Ana Maria Rodrigues, Jaime Da Cunha Branco, Helena Canhão, Susan L. Hughes, Constança Paúl

**Affiliations:** ^1^Research Unit on Ageing, Instituto de Ciências Biomédicas Abel Salazar (ICBAS), Centro de Investigação em Tecnologias e Serviços de Saúde (CINTESIS), University of Porto, Porto, Portugal; ^2^EpiReumaPt Study Group—Sociedade Portuguesa de Reumatologia, Lisboa, Portugal; ^3^EpiDoc Unit—Unidade de Epidemiologia em Doenças Crónicas (CEDOC, NMS/UNL), Unidade de Investigação em Reumatologia, Instituto de Medicina Molecular, Faculdade de Medicina da Universidade de Lisboa, Lisboa, Portugal; ^4^EpiReumaPt Study Group—Centro de Estudos de Doenças Crónicas (CEDOC) da NOVA Medical School, Universidade Nova de Lisboa (NMS/UNL), Centro Hospitalar Lisboa Ocidental (CHLO-EPE), Sociedade Portuguesa de Reumatologia, Lisbon, Portugal; ^5^EpiReumaPt Study Group—Centro de Estudos de Doenças Crónicas (CEDOC), EpiDoC Unit, NOVA Medical School, Universidade Nova de Lisboa (NMS/UNL), Escola Nacional de Saúde Publica, Universidade Nova de Lisboa, Sociedade Portuguesa de Reumatologia, Serviço de Reumatologia do Hospital de Santa Maria, Centro Hospitalar Lisboa Norte (CHLN-EPE), Lisboa, Portugal; ^6^Community Health Sciences, School of Public Health, Center for Research on Health and Aging, University of Illinois at Chicago, Chicago, IL, United States

**Keywords:** predictors, rheumatic disease, aging, disability, comorbidity, women

## Abstract

**Objective:**

This study aimed to identify independent associations of sociodemographic, functionality, physical activity, physical and mental health, and osteoarthritis (OA), among older adults.

**Methods:**

A sample of 1,645 older adults (50+ years) observed by rheumatologists, from EpiReumaPt, a population-based study was analyzed. A structured interview included sociodemographic data, chronic non-communicable disease, and physical activity. Functional ability was assessed by the Health Assessment Questionnaire Disability Index; depression and anxiety were assessed by Hospital Anxiety and Depression Scale. OA (knee OA and/or hip OA and/or hand OA) was defined after medical evaluation by rheumatologists according to expert opinion combined with the fulfillment of the American College of Rheumatology classification criteria.

**Results:**

1,059 participants (64.9%) met the OA classification criteria. Statistically significant differences were found between persons with and without OA in all sociodemographic variables, non-communicable diseases, functional status, physical activity, depression, and anxiety. In the unadjusted logistic regression models, all variables were associated with OA. The final adjusted model explained 32% of the variance. Those who are female with higher age, have more than five comorbidities, and lower levels of function and physical activity were more likely to meet the criteria for a diagnosis of OA.

**Discussion:**

We have analyzed data from a population-based study and found that a diagnosis of OA was independently associated with age, female gender, higher number of comorbidities, physical disability, and low levels of physical activity. These results reinforce the usefulness of the development of a multidimensional assessment to design and test effective interventions for this population.

## Introduction

Osteoarthritis (OA) is the most prevalent rheumatic disease in the general population (1), and its prevalence is known to increase gradually with age (2). The World Health Organization (WHO) Scientific Group on Rheumatic Diseases estimates that 10% of the world’s population 60 years or older has significant clinical problems that can be attributed to OA (3).

Osteoarthritis is a major contributor to functional impairment and loss of independence in older persons causing difficulties in maintaining their activities of daily living (4). Data from the *Global Burden of Disease Project* identify the common causes of years of healthy life lost due to disability in people older than 60 years and identify OA as one of the 10 most relevant causes of disability in this population (5).

Because of the high prevalence of this disease and its impact on independence and quality of life among patients, researchers have been studying factors associated with OA prevalence. Sociodemographic variables, such as age and gender, have been the most studied factors, but studies have also analyzed the association of OA with comorbidities [e.g., Ref. (6, 7)], psychological factors (8, 9), and physical activity (10, 11).

Currently, few studies have compared people with and without OA, furthermore, most studies have analyzed associated factors separately and the comparative contribution of each variable is unknown.

The objectives of this study are (1) to compare people with and without OA with respect to sociodemographic characteristics, functionality, physical activity, non-communicable chronic diseases, and presence of anxiety and depression symptoms; and (2) to identify independent associations between sociodemographic characteristics, functionality, physical activity, physical health and psychological symptoms, and OA.

## Materials and Methods

### Sample

Data from EpiReumaPt project, which is a national, cross-sectional, and population-based study, were used (12). This study population comprised adults (≥18 years old) living in the community, in the Portugal mainland and islands (Azores and Madeira). Exclusion criteria were resident of an institution, and inability to speak Portuguese or to complete the assessment protocol. Participants were selected through a process of multistage random sampling. The sample was stratified by region and population size.

EpiReumaPt involved a three-stage approach. The first stage included the selection of potential participant household/families using a random selection process. In each household, the adult with the most recent birthday was recruited and completed a structured interview (sociodemographic, socioeconomic, anxiety and depression symptoms, lifestyle habits, chronic non-communicable diseases, health resources utilization data), and a screening for rheumatic diseases. In the second stage, all participants who screened positive for at least one rheumatic disease plus 20% of participants with no rheumatic symptoms were invited to be assessed by a rheumatologist. In the last phase, a team of three experienced rheumatologists revised all the clinical, laboratory, and imaging data and confirmed the diagnoses using validated criteria (12). Details of the EpiReumaPt methodology have been published previously (12–14).

In this study, the sample consisted of adults 50+ years old who participated in the second phase of EpiReumaPt study and were evaluated by a rheumatologist to confirm the presence or absence of OA (knee OA and/or hip OA and/or hand OA). OA was defined by rheumatologists according to expert opinion combined with the fulfillment of the American College of Rheumatology classification criteria for knee OA, hip OA, and hand OA, as described earlier (15–17).

Sociodemographic data, physical function, anxiety and depression symptoms, lifestyle (e.g., physical activity), and chronic non-communicable diseases were collected during the first stage of the EpiReumaPt project.

### Measurements

Sociodemographic data included age, gender, marital status, education, and work status. Marital status was categorized in three categories: without partner (single and divorced people), married, and widower. Work status was categorized as employed full time, part-time, not currently employed (domestic employed, unemployed, and students), and retired.

Number of current chronic non-communicable diseases was assessed using a comprehensive list which included the following 14 diseases: high blood pressure, high cholesterol, heart disease, diabetes mellitus, chronic lung disease, problems in the digestive tract, renal colic, neurological disease, allergies, mental or psychiatric illness, cancer, thyroid and parathyroid problems, hypogonadism, hyperuricemia, and other rheumatic diseases (OA was excluded from this list).

The Health Assessment Questionnaire Disability Index [HAQ-DI; (18)] was used to assess level of functional ability. HAQ-DI measures difficulties with the performance of 20 daily activities retrospectively over the preceding week with four possible responses: no difficulty (0 points), some difficulty (1 point), much difficulty (2 points), and unable to do (3 points). The 20 activities are classified into 8 categories with 2 or 3 activities each: dressing and grooming, arising eating, walking, personal hygiene, reaching, gripping, and usual activities (i.e., shopping, doing chores, getting in and out of a car) (18, 19). The score for each category is the single response within the category with the highest score (greater difficulty). The total score is computed as the mean of the eight category scores. As in previous studies, we did not correct HAQ-DI scores for use of assistive devices (19, 20).

To assess physical activity, participants were asked about the amount of time (minutes) per week they dedicated to physical activity. Answers were categorized as <150 or ≥150 min, based on the physical activity recommendations of the WHO (21) for older adults.

Depression and anxiety were assessed by Hospital Anxiety and Depression Scale [HADS; (22, 23)]. The HADS is a short instrument with two subscales that both have seven items that assess presence of clinical anxiety and depression. The scale has a maximum score of 21 for both anxiety and depression subscales. People with scores between 0 and 7 are considered normal, scores between 8 and 10 are borderline, and a score ≥11 indicates a mood disorder. In data analysis, anxiety and depression were considered as dichotomous variables (i.e., a participant with a score ≥11 on either subscale was considered depressed or anxious).

### Ethical Issue

EpiReumaPt was approved by the National Committee for Data Protection and by NOVA Medical School Ethics Committee. All participants provided informed consent, and the study was performed according to the principles established by the Declaration of Helsinki.

### Statistical Analysis

Participants (*N* = 1,645) were divided in two groups: group 1 (*n* = 1,059) consisted of participants meeting the OA diagnostic criteria; and group 2 (*n* = 586) consisted of participants without OA diagnosis or others rheumatic diseases. There were no missing data except for physical activity (*n* = 485).

Descriptive analysis was used to describe the main characteristics of the sample, using frequencies (absolute and relative) or means and SDs, depending on the type of the variable. The comparison of groups defined by the presence/absence of OA (group 1: with OA and group 2: without OA) was performed using independent samples *t*-test for continuous variables and Chi-square test for categorical variables. Univariate logistic regression models, with presence of OA as the outcome, were performed to identify a set of factors that could predict OA presence, including sociodemographic characteristics, comorbidities, functional status, physical activity, depression, and anxiety. The final multivariable model with backward elimination included variables with *p*-values <0.05. Odds ratios (ORs) and 95% confidence intervals were used to assess the results. All statistical analyses were performed using Statistical Package for the Social Sciences (SPSS, version 24, IBM), and α = 0.05 was used to determine significance level.

## Results

### Characteristics of the Sample

The sample comprised 1,645 participants. The mean age of the sample was 65.3 years (SD = 9.3) and 63.2% (*n* = 1,040) were women. Most of the participants were married (*n* = 1097, 66.7%) and had 4 years of education (*n* = 847, 54.5%). Regarding work status, the majority were retired (*n* = 1050, 63.8%). With respect to number of self-reported chronic non-communicable diseases, most participants reported 0–2 (*n* = 720, 43.8%) and 3–4 (*n* = 540, 32.8%) chronic diseases. Hypertension (*n* = 857, 52.1%) and high cholesterol (*n* = 805, 48.9%) were the most frequently reported diseases. Among rheumatic diseases, osteoporosis was the most frequently self-reported disease (*n* = 190, 11.6%). With respect to functional status, the mean score of HAQ-DI for the total sample was 0.55 (SD = 0.65) out of 3, indicating low levels of limitations. The majority of the sample met the ≥150 min of physical exercises per week (*n* = 306; 63.1%). Depressive symptoms were found in 10.6% (*n* = 174) and anxiety was found in 13.8% (*n* = 227) of the total sample.

Sample consisted of 1,059 adults with OA diagnosis (64.4%) and 586 without OA diagnosis (35.6%). Among people with OA, 12.5% (*n* = 132) were classified as having hip OA, 44.7% (*n* = 473) hand OA, and 68.6% knee OA (*n* = 726). Among persons with an OA diagnosis, 36.6% (*n* = 388) reported the presence of other rheumatic diseases. In this sample, the additional rheumatic diseases most commonly reported were osteoporosis (*n* = 190, 11.6%) and rheumatoid arthritis (*n* = 91, 5.5%).

Statistically significant differences were found between groups (with and without OA) in all sociodemographic variables (i.e., age, gender, marital status, education, and work status), non-communicable diseases, functional status, physical activity, depression, and anxiety (Table 1). Participants with OA were older (mean = 67.1, SD = 9.1) than participants without OA (mean = 62.1, SD = 8.9). The proportion of women with OA (*n* = 774, 73.1%) was higher than among persons without OA (*n* = 266; 45.4%). More participants with OA were married (*n* = 666, 63.5%), in comparison to persons without OA (*n* = 425, 72.5%). Widowers were the second most common category represented, 263 (24.8%) in participants with OA compared to 71 (12.1%) in those without OA. Regarding work status, the majority of participants in both groups were retired (persons with OA: *n* = 749, 70.7%; persons without OA: *n* = 301, 51.4%). Among participants with OA, the second most frequent category was “without labor activity” (*n* = 158, 14.9%), whereas for persons without OA, the second most common category was “full time employed” (*n* = 176, 30.0%). Most persons with OA reported presence 3–4 non-communicable diseases (*n* = 367, 34.7%), compared to 0–2 non-communicable diseases (*n* = 365, 62.3%) among persons without OA. Individuals with OA were significantly less active (56.8% met the recommendation of ≥150 min of physical activity per week) than those without OA (70.8% met the recommendation of ≥150 min of physical activity per week). With respect to functional status, participants with OA showed significantly higher levels of functional limitations (mean = 0.70, SD = 0.67) than persons without OA (mean = 0.28, SD = 0.58). Percentage of depressive symptoms was also higher among individuals with OA (*n* = 143, 13.5%) compared to participants without OA (*n* = 93; 8.7%). Similar results were found for anxiety, persons with OA showed a higher percentage of anxiety symptoms (*n* = 172, 13.8%) than people without OA (*n* = 55, 9.4%).

**Table 1:** Sociodemographic and health description of the sample and comparison of groups [with and without osteoarthritis (OA) diagnosis].

	Total (*n* = 1,645)	With OA (*n* = 1,059)	Without OA (*n* = 586)	*p*
	*n* (%)	*n* (%)	*n* (%)	
**Age (years)**^a^	65.3 (9.3)	67.1 (9.1)	62.1 (8.9)	<0.001
**Gender**					
Male	605 (36.8)	285 (26.9)	320 (54.6)	<0.001
Female	1,040 (63.2)	774 (73.1)	266 (45.4)	
**Marital status**					
Without partner	214 (13.0)	124 (11.7)	90 (15.4)	<0.001
Married	1,097 (66.7)	666 (63.5)	425 (72.5)	
Widower	334 (20.3)	263 (24.8)	71 (12.1)	
**Education**					
0–3 years	242 (14.7)	194 (18.3)	48 (8.2)	<0.001
4 years	847 (54.5)	580 (54.8)	267 (45.6)	
5–9 years	274 (16.7)	140 (13.2)	134 (22.9)	
10+ years	282 (17.1)	145 (13.7)	137 (23.4)	
**Work status**										
Full time employed	295 (17.9)	119 (11.2)	176 (30.0)	<0.001
Partial time employed	52 (3.2)	33 (3.1)	19 (3.2)	
Without labor activity	248 (15.1)	158 (14.9)	90 (15.4)	
Retired	1,050 (63.8)	749 (70.9)	301 (51.4)	
**Non-communicable diseases**					
0–2	720 (43.8)	355 (33.5)	365 (62.3)	<0.001
3–4	540 (32.8)	367 (34.7)	173 (29.5)	
5 or +	385 (23.4)	337 (31.8)	48 (8.2)	
**Physical disability^a^ (HAQ)**	0.55 (0.65)	0.70 (0.67)	0.28 (0.53)	<0.001
**Physical activity per week (*n* = 485)**										
<150 min	179 (36.9)	115 (43.2)	64 (29.2)	0.001
≥150 min	306 (63.1)	151 (56.8)	155 (70.8)	
**Depressive symptoms**					
Present	174 (10.6)	143 (13.5)	31 (5.3)	<0.001
Non-present	1,471 (89.4)	916 (86.5)	555 (94.7)	
**Anxiety symptoms**				
Present	227 (13.8)	172 (16.2)	55 (9.4)	<0.001
Non-present	1,418 (86.2)	887 (83.8)	531 (90.6)	

*^a^Mean (SD)*.

### Multivariate Predictors of OA

Using OA as the outcome, unadjusted logistic regression models found that all of the variables shown in Table 1 were associated with presence of this chronic disease (Table 2). The final adjusted model contains only significant variables, and the non-significant variables were excluded, one by one, from the model. The variables were removed in the following sequence: marital status (*p* = 0.387), work status (*p* = 0.216), depression (*p* = 0.512), and anxiety (*p* = 0.066). In the final adjusted model, age, gender, number of comorbidities, physical disability, and physical activity remained statistically significant and the model explained 32% of the variance. People who were older (OR = 1.048, *p* < 0.001) and female (OR = 3.192, *p* < 0.001) were more likely to have OA. People with more than five comorbidities were more likely to report OA than people with 0–2 comorbidities (OR = 4.484, *p* < 0.001). Functional disability was also associated with OA. Specifically, persons with higher levels functional disability were more likely to report OA than those with lower levels of disability (OR = 1.926, *p* < 0.001). Physical activity was also associated with OA persons; people with lower levels of physical activity were more likely to report OA than those with higher levels of physical activity (OR = 1.630, *p* = 0.027).

**Table 2:** Associations of potential explanatory variables with osteoarthritis (OA).

			Unadjusted			Adjusted		
	*N*	Cases of OA	Odds ratio (OR)	95% confidence intervals (CI 95%)	*p*	OR	CI 95%	*p*
**Age**	1,645	67.1 (9.1)	1.063	1.051–1.076	<0.001	1.048	1.021–1.076	<0.001
**Gender**					<0.001			<0.001
Male	605	285 (26.9%)	1	–	–	1	–	–
Female	1,040	774 (73.1%)	3.269	2.643–4.038	<0.001	3.192	2.040–4.994	<0.001
**Marital status**					<0.001	–	–	–
Without partner	214	124 (11.7%)	1	–	–			
Married	1,097	672 (63.5%)	1.148	0.852–1.545	0.364			
Widower	334	263 (24.8%)	2.689	1.844–3.921	<0.001			
**Education**					<0.001			0.037
≥10 years	282	145 (13.7%)	1	–	–	1	–	–
5–9 years	274	140 (13.2%)	0.975	0.708–1.377	0.939	0.583	0.321–1.059	0.076
4 years	847	580 (54.8%)	2.052	1.559–2.702	<0.001	1.182	0.715–1.956	0.515
0–3 years	242	194 (18.3%)	3.819	2.578–5.656	<0.001	0.503	0.192–1.313	0.160
**Work status**					<0.001	–	–	–
Full time employed	295	119 (11.2%)	1	–	–			
Partial time employed	52	33 (3.1%)	2.569	1.395–4.730	0.002			
Without labor activity	248	158 (14.9%)	2.596	1.833–3.677	<0.001			
Retired	1,050	749 (70.9%)	3.814	2.814–4.813	<0.001			
**Comorbidities**					<0.001			<0.001
0–2	720	355 (33.5%)	1	–	–	1	–	–
3–4	540	367 (34.7%)	2.181	1.729–2.752	<0.001	2.850	1.801–4.511	<0.001
5 or more	385	337 (31.8%)	7.219	5.160–10.099	<0.001	4.484	2.348–8.561	<0.001
**Physical disability (HAQ)**	1,645	0.70 (0.67)^a^	3.871	3.081–4.865	<0.001	1.926	1.186–3.126	<0.001
**Physical activity per week**					0.002			0.027
≥150 min	306	151 (56.8%)	1	–	–	1	–	–
<150 min	179	115 (43.2%)	1.844	1.263–2.694	0.002	1.630	1.056–2.516	0.027
**Depression**					<0.001	–	–	–
No	1,471	916 (86.5%)	1	–	–			
Yes	174	143 (13.5%)	2.795	1.869–4.179	<0.001			
**Anxiety**					<0.001	–	–	–
No	1,418	887 (83.8%)	1	–	–			
Yes	227	143 (16.2%)	1.872	1.357–2.584	<0.001			

*^a^Mean (SD)*.

## Discussion

The results from this study showed a positive association between older age, female gender, comorbidities (higher number of non-communicable diseases), disability, physical activity level, and presence of OA; findings that are consistent with previous results reported in the literature on OA. According to *A National Public Health Agenda for Osteoarthritis* (24), OA prevalence increases substantially at age 45. Also, the *World Health Organization World Report on Ageing and Health* (25) showed a higher rate of knee and hip OA in people above 65 years and in females. Women reported higher percentage of OA than men; these results highlight the importance of considering gender differences among people with OA. We developed an exploratory analysis of this same sample, and the results showed that women had lower levels of education, more non-communicable diseases, higher levels of disability, and depressive and anxiety symptoms than men. These results indicate that interventions that seek to improve function among persons with OA should consider the profile of the largely female population of persons with OA to maximize adherence to and impact of interventions in this population.

Other sociodemographic variables tested: marital status, and work status, lost their significance in the adjusted model, probably because people with OA and with low levels of education are mostly women (*n* = 554, 52.3%) and had higher levels of physical activity (*n* = 73, 15.1%), furthermore, retired people was the oldest group (mean age = 70.9, SD = 7.4) and had the highest level of disability (mean = 0.77, SD = 0.69). Prior studies have analyzed the impact of sociodemographic characteristics in OA, for example, Cleveland et al. (26) explored the relationship between functional status/disability and sociodemographic status in people with OA and found that persons with lower education, non-homeowners, or lower income were more likely to have increased disability.

Persons with OA seem to have a higher number of others non-communicable diseases. This result is consistent with a systematic review on multimorbidity which showed that depression, hypertension, diabetes, arthritis, asthma, and OA were prone to be comorbid with other conditions (7).

We found a positive relationship between physical disability and OA. The mean disability score, in this study among persons with OA, was 0.70, higher than the mean score that was reported for a general population (0.49) (19), but very similar to the mean reported in a population with OA (0.80) (19). The same authors have stated referred that HAQ-DI scores of 0–1 are generally considered to represent mild to moderate difficulty functioning. According to the WHO (27), among people with OA, 80% had mobility disability and 25% were unable to perform activities of daily living independently. On the one hand, OA appears to have a high impact in disability; on the other hand, low levels of functioning promote sedentary behavior which is a risk factor for OA as well as for weight gain, general de-conditioning, and possibly the onset of other chronic conditions.

Our results showed that 56.8% of people with OA met the physical activity recommendations. This percentage is lower than the level of physical activity performed by people without OA, suggesting that the presence of OA can be a barrier to physical activity participation. The *National Food, Nutrition and Physical Activity Survey* Report (28) found low levels of physical activity in the general Portuguese population. In this national report, only 36% of young adults (15–21 years), 27% of adults, and 22% of older adults (65–84 years) met the actual recommendations for physical activity. This issue is particularly important for adults with OA, because this group is physically inactive and at risk of disability due to joint limitations (4). Several studies confirm the importance of physical activity for arthritis and have shown that increased physical activity is associated with improved disability in people with arthritis and in OA symptomatology relief (4, 25, 29). International organizations recognize regular physical activity as one of the most important non-pharmacological therapies for OA (30–32).

One study that explored the physical activity levels in knee OA patients concluded that only 41% of patients reported sufficient levels of physical activity (33). Badley and Ansari (10) concluded that physical inactivity accounted for an estimated 21% of disability attributed to arthritis and randomized clinical trials demonstrated that structured physical activity can reduce the risk of developing disability in people with OA (34).

In this study, symptoms of depressive and anxiety symptoms were higher in people with OA than in people without OA. However, in the adjusted model, these two variables ceased to be significant. According to Eurostats (35), in 2014, 7.1% of the population of EU-28 reported having chronic depression. Portugal with 11.9% was top ranked in the share of total population reporting chronic depression. Stubbs et al. (9) concluded that in Europe there is a prevalence of 19.9% of depressive symptoms in people with OA, lower than the prevalence found in studies conducted in USA (23.1%). Few studies have analyzed the symptoms of anxiety among OA patients, as most of the studies focused in the rheumatic disease in general [e.g., Ref. (36)].

Despite the results, study of psychological morbidity continued among people with OA remains important because it can affect the diagnosis and the adherence to treatment (36–38). Depressive symptoms appear to act as a potential barrier to physical activity and social participation for people with OA (9).

This study has some limitations, namely, the cross-sectional nature of the data; and the missing information about the time of duration of OA and pain, which could help us to understand the relationship between specific variables like physical activity and disability. Physical activity was assessed in a reduced number of participants which could limit the interpretation of the results. Those without a physical activity assessment could be more inactive than those who filled all the evaluation. The prevalence of OA is overestimated in this sample when compared with the Portuguese population (50+ years). This aspect is related to the general objectives of EpiReumaPt that focus on people with rheumatic diseases and with the criteria for sample selection in this study. The amount of variance in OA explained by the model was low (32%), which limits the interpretation of the results. The variance explained could be improved by the inclusion of other biological variables, such as body mass index and muscle strength. Other limitations of the EpiReumaPt initiative were reported by Branco et al. (12). The main strengths of the study are first the large national population-based sample and second, the fact that the OA diagnosis was made by rheumatologists who are experts in performing these evaluations. A final strength of the dataset and design is that it enabled a systematize comparison of the characteristics of person with and without OA using a large number of sociodemographic, disease, and functional status variables.

In this paper, we have analyzed older adults (+50) from a population-based study and found that a diagnosis of OA was independently associated with age, female gender, higher number of comorbidities, physical disability, and low levels of physical activity. These results reinforce the need to of the develop multidimensional assessments to design and evaluate effective interventions for this population.

## Ethics Statement

EpiReumaPt was approved by the National Committee for Data Protection and by NOVA Medical School Ethics Committee. All participants provided informed consent, and the study was performed according to the principles established by the Declaration of Helsinki.

## Author Contributions

ND contributed to the conception, design, analysis, and interpretation of data for the work. She agreed to be accountable for all aspects of the work in ensuring that questions related to the accuracy or integrity of any part of the work are appropriately investigated and resolved. AR, JB, HC, and SH revised the work critically for important intellectual content and approved the final version to be published. CP contributed to the design, analysis, and interpretation of the data for the work; revised the work critically for important intellectual content; and approved the final version to be published.

## Conflict of Interest Statement

All co-authors have seen and agree with the contents of the manuscript and there is no financial interest to report. We certify that the submission is original work and is not under review at any other publication. No previous data have been reported before. All co-authors confirm the full control of all primary data and we agree to allow the journal to review their data if requested.
